# Outcome of half-dose photodynamic therapy in chronic central serous chorioretinopathy with fovea-involving atrophy

**DOI:** 10.1007/s00417-020-04959-3

**Published:** 2020-10-29

**Authors:** Thomas J. van Rijssen, Elon H. C. van Dijk, Paula Scholz, Robert E. MacLaren, Sascha Fauser, Susan M. Downes, Carel B. Hoyng, Camiel J. F. Boon

**Affiliations:** 1 Department of Ophthalmology, Leiden University Medical Center, P.O. Box 9600, 2300 RC Leiden, The Netherlands; 2 Department of Ophthalmology, University Hospital of Cologne, Cologne, Germany; 3 Oxford Eye Hospital, John Radcliffe Hospital, Oxford University Hospitals NHS Foundation Trust, West Wing, Oxford, UK; 4 F. Hoffmann-La Roche, Basel, Switzerland; 5 Department of Ophthalmology, Radboud University Medical Center, Nijmegen, The Netherlands; 6 Amsterdam University Medical Centers, Academic Medical Center, Department of Ophthalmology, University of Amsterdam, Amsterdam, The Netherlands

**Keywords:** Central serous chorioretinopathy, Foveal atrophy, Photodynamic therapy, Fluorescein angiography

## Abstract

**Purpose:**

To evaluate the clinical outcomes after half-dose photodynamic therapy (PDT) in chronic central serous chorioretinopathy (cCSC) patients with pre-existent fovea-involving atrophy.

**Methods:**

In this retrospective study, cCSC patients who had a window defect of the retinal pigment epithelium (RPE) on fluorescein angiography (FA), compatible with RPE atrophy, prior to half-dose PDT were included.

**Results:**

Thirty-four cCSC eyes with typical findings of cCSC on multimodal imaging, and fovea-involving RPE atrophy on FA, were included. At the first visit after PDT (at a median of 1.8 months after half-dose PDT), 20 eyes (59%) had a complete resolution of SRF (*p* < 0.001), while this was the case in 19 eyes (56%) at final visit (median of 11.3 months after half-dose PDT; *p* < 0.001). The mean BCVA in Early Treatment of Diabetic Retinopathy Study letters was 71. 2 ± 15.9 at last visit before PDT, which increased to 74.1 ± 14.1 at first visit after PDT (*p* = 0.093, compared with baseline), and changed to 73.0 ± 19.1 at final visit (*p* = 0.392, compared with baseline). Both at first visit after PDT and at final visit, a significant decrease in subfoveal choroidal thickness was observed (*p* = 0.032 and *p* = 0.004, respectively).

**Conclusions:**

Half-dose PDT in cCSC patients with pre-existing fovea-involving atrophy may lead to anatomical changes, but not to functional improvements. Ideally, cCSC should be treated with half-dose PDT before the occurrence of such atrophy.

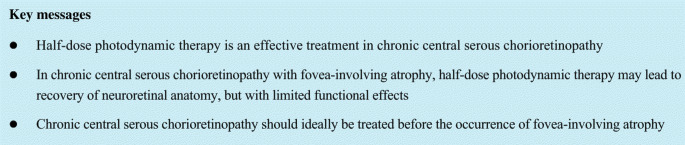



## Introduction

Central serous chorioretinopathy (CSC) is a common macular disease in which a serous detachment of the neuroretina occurs, often in the macula, with subsequent vision loss [1]. The disease usually presents with visual symptoms including decreased visual acuity, diminished contrast vision and/or metamorphopsia. Middle-aged men are most often affected, while the use of corticosteroids has been described to be the most important exogenous risk factor for the development of CSC [2]. Although the exact aetiology of CSC is unknown, the choroid is presumed to be primarily affected. Choroidal congestion and hyperpermeability have been found to induce damage to the retinal pigment epithelium (RPE) which may lead to atrophic RPE abnormalities (pachychoroid pigment epitheliopathy). In typical CSC, accumulation of subretinal fluid (SRF) occurs when fluid leaks through a defect in the outer blood-retinal barrier of the RPE [3]. Multimodal imaging consisting of optical coherence tomography (OCT), fluorescein angiography (FA), indocyanine green angiography (ICGA), are pivotal for diagnosing CSC and OCT angiography can aid in detecting subretinal neovascularisation in cases of CSC or diseases mimicking CSC. While OCT is important to detect SRF, FA is of additional value since it provides information on leakage sites and the extent of atrophic RPE alterations. Using ICGA, the extent of underlying choroidal abnormalities can be assessed. Based on the findings on multimodal imaging and the duration of symptoms, CSC is often categorized into acute CSC and chronic CSC (cCSC), although the classification is subject of debate [4]. While acute CSC usually resolves spontaneously within 4 months after the start of symptoms, cCSC can lead to irreversible visual impairment and a decreased quality of life [5, 6]. For cCSC, several subtypes have been described including for example cCSC with focal or diffuse leakage on FA, severe cCSC cases with diffuse atrophic RPE alterations and/or cCSC with posterior cystoid retinal degeneration (PCRD) [7–10]. Treatment in cCSC patients is recommended in order to reduce the duration of the serous detachment, to improve vision and to reduce the recurrence rate [5, 11, 12]. The first large randomized controlled trial in cCSC, the PLACE trial, reported superiority of half-dose PDT over treatment with high-density subthreshold micropulse laser [13]. Currently, most evidence on efficacy is available for half-dose (or half-fluence) photodynamic therapy, which is therefore considered to be the treatment of choice for cCSC [11].

Retrospective data suggest that PDT may be the best option also in severe cases of cCSC, with multifocal leakage points, more extensive RPE atrophy and/or PCRD, but robust data on the outcome of treatment in this severe end of the disease spectrum are scarce [9, 10]. The presence of (foveal) RPE atrophy in cCSC may be suggestive of a prolonged serous detachment and/or RPE and choroidal dysfunction and is associated with a worse visual outcome [5, 14, 15]. However, no clear definition exists and no data on treatment outcome are available in this specific subgroup of cCSC patients. While half-dose and half-fluence PDT in uncomplicated cCSC are highly effective and safe, it is unknown if the presence of fovea-involving atrophy may influence the efficacy and visual outcome of PDT in cCSC, which may in turn have important clinical implications with regard to counselling. The aim of this study is to report the outcome of PDT in cCSC patients with pre-existent fovea-involving RPE atrophy.

## Methods

In this retrospective study, cCSC patient records and multimodal imaging between November 2012 and August 2019 were studied. Four academic medical centres located in Cologne (Germany), Oxford (United Kingdom), and Leiden and Nijmegen (the Netherlands) participated in this study. The institutional review board waived the need for approval from the medical ethical committee because of the retrospective nature of this study (register number: G19.093). This study adhered to the tenets of the Declaration of Helsinki.

**Fig. 1:**
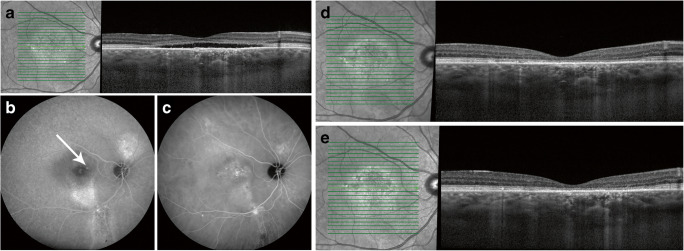
Multimodal imaging of a patient diagnosed with chronic central serous chorioretinopathy and fovea-involving atrophy before half-dose photodynamic therapy (PDT; **a**–**c**), at the first visit 2 months after PDT (**d**), and at 12 months after PDT (**e**). First visual complaints were reported 18 months before half-dose PDT. At the visit before PDT, foveal subretinal fluid is visible on optical coherence tomography (**a**). Foveal leakage of fluorescein and retinal pigment epithelium alterations were observed on fluorescein angiography (FA; white arrow, **b**), along with changes in the degree of fluorescence on indocyanine green angiography (ICGA; **c**). The central retinal thickness changed from 70 μm before PDT, to 81 μm at first visit after PDT, and to 59 μm at final visit. Despite the resolution of subretinal fluid after PDT, best-corrected visual acuity decreased from 76 Early Treatment of Diabetic Retinopathy Study (ETDRS) letters at last visit before PDT to 72 ETDRS letters at first visit after PDT and to 70 ETDRS letters at final visit

All included patients had a subjective loss of vision for at least 3 months, which was interpreted as the onset of CSC. The following imaging characteristics were mandatory to be eligible for inclusion: SRF affecting the fovea on spectral-domain OCT, one or more regions of active leakage combined with RPE window defects visible on FA and hyperfluorescent changes typical of cCSC visible on ICGA. In addition, eligible patients had to have diffuse atrophic RPE alterations that included the fovea on FA at baseline, as evidenced by a granular RPE window defect on mid-phase FA (Fig. 1) [9], and had received half-dose PDT. Patients with evident choroidal neovascularisation, other causes than cCSC of a decreased visual acuity, or who previously received PDT in another centre were excluded. The presence of PCRD at baseline and/or current or previous corticosteroid use were not considered to be exclusion criteria. The inclusion and exclusion criteria are summarized in Table 1.

**Table 1:** Inclusion and exclusion criteria of chronic central serous chorioretinopathy patients with fovea-involving atrophy

Inclusion criteria	Exclusion criteria
Male or female cCSC patients ≥18 years old	Presence of soft drusen or signs of neovascularisation
Visual loss and/or presence of SRF on OCT > 6 weeks	Evidence of another diagnosis that could explain either the vision loss or the SRF
SRF on OCT	Previous PDT treatment in another centre, with insufficient information available
Presence of foveal atrophic RPE alterations on FA and presence of choroidal hyperfluorescence on ICGA	

*(c)CSC* (chronic) central serous chorioretinopathy, *FA* fluorescein angiography, *ICGA* indocyanine green angiography, *OCT* optical coherence tomography, *PDT* photodynamic therapy, *SRF* subretinal fluid

Fovea-involving atrophy was defined as the presence of granular foveal hyperfluorescence due to a RPE window defect on mid-phase FA, without evidence of subretinal neovascularisation. These findings on FA are accompanied by a relatively thin neuroretinal thickness on OCT (Fig. 1) and can also be referred to as diffuse atrophic RPE alterations. Cases were collected and graded by one of the authors (TJVR) and discussed with an experienced retina specialist (CJFB) in order to ascertain the presence of fovea-involving atrophy.

Data on age, gender, ethnicity, previous CSC treatment(s), use of corticosteroids and duration of symptoms were obtained at last visit before PDT. At the time of PDT, the fluency, duration of laser treatment and verteporfin dose that were used were collected. Multimodal imaging including spectral-domain (SD) OCT, FA and ICGA was obtained with the Heidelberg Spectralis device (Heidelberg Engineering, Heidelberg, Germany). The best-corrected visual acuity (BCVA), presence of SRF, central foveal thickness (CFT), subfoveal choroidal thickness (SFCT), integrity of the external limiting membrane (ELM) and integrity of the ellipsoid zone (EZ) were obtained at the last visit before PDT, at the first visit after PDT and at the final visit. The CFT was defined as the distance between the inner borders of the internal limiting membrane and the inner border of the ellipsoid zone, which was measured manually on spectral-domain OCT according to a previously described method to ensure that SRF is not incorrectly included in the CFT measurement [16]. SFCT was measured as the distance between the outer border of the RPE and the inner border of the choroid-sclera junction on enhanced depth imaging spectral-domain OCT. The built-in calliper in the Heidelberg OCT machine software was used to measure both the CFT and SFCT. The integrity of the ELM and EZ was defined as continuous when there was no interruption or significant decrease in signal intensity of either the ELM or EZ within the foveal area on OCT.

### PDT procedure

First, the eye in need of treatment was dilated with the use of a mydriatic agent. Consequently, verteporfin (Visudyne®; Novartis, Basel, Switzerland) with 3 mg/m^2^ (half dose) was administered intravenously within a 10-minute time frame. After the administration of verteporfin, the eye in need of treatment was anaesthetised and a PDT magnification lens was positioned on the eye. Then, at exactly 15 min after the start of infusion of verteporfin, light with a wavelength of 689 nm and a full fluency of 50 J/cm^2^ was applied to the area in need of treatment (hyperfluorescent areas typical of cCSC on mid-phase ICGA) within 83 s.

### Statistical analyses

Analyses were performed with SPSS Statistics (IBM Corp. version 23.0. Armonk, New York, USA). In case of a normal distribution, both *t* tests and Chi-square tests were used. When data were skewed, Mann-Witney tests or Wilcoxon signed rank tests were used. *P* values <0.05 were deemed statistically significant.

## Results

Between November 2012 and September 2019, 628 medical records of CSC patients were screened. A total of 34 eyes of 32 cCSC patients with fovea-involving atrophy, including 30 men and 2 women, were included in this study. Thirty-three patients were of Caucasian descent and one patient was of non-Caucasian descent. The mean age was 57.4 ± 10.8 years at last visit before PDT. The duration of visual symptoms varied from 3 to 191 months (median: 9 months). Current corticosteroid use was reported in 1 patient and previous corticosteroid use was reported in 3 out of 32 patients. Diffuse atrophic RPE alterations were present also outside the fovea in 22 eyes (64%). The time between last visit before PDT and PDT varied from 1 to 57 days, with a median of 21 days. The number of days between PDT and first visit after PDT ranged from 15 to 203 days, with a median of 53 days. The median duration from PDT until last available visit was 11.3 months, with a range from 1 month to 4.4 years. A total of 13 eyes had received treatment(s) prior to half-dose PDT. Out of these eyes, three eyes had previously received intravitreal anti-vascular endothelial growth factor treatment, eight eyes were previously treated with high-density subthreshold micropulse laser, and two eyes were treated with oral eplerenone. There were seven eyes that received a second half-dose PDT between the first PDT and last available visit. There was one eye that received a total of three PDTs between last visit before first PDT and last available visit.

By definition, SRF was present in all eyes at last visit before PDT. In 20 eyes (59%), SRF had completely resolved at first visit after PDT (*p* < 0.001, compared with baseline), and at final visit, SRF had completely resolved in 19 eyes (56%; *p* < 0.001, compared with baseline). The number of eyes with PCRD was four (13%) at last visit before PDT, and this decreased to two eyes (8%) at first visit after PDT. At final visit, PCRD remained present in two eyes. The foveal ELM was intact on SD-OCT in 5 eyes (15%) at last visit before PDT, while this increased to 12 eyes (35%) at first visit after PDT (*p* = 0.065, compared with baseline) and to 13 eyes (38%) at final visit (*p* = 0.039, compared with baseline). The foveal EZ was intact in three eyes (9%) at last visit before PDT and increased to eight eyes (24%) at first visit after PDT (*p* = 0.125, compared with baseline) and to nine eyes (26%) at final visit (*p* = 0.031, compared with baseline). There was a decrease in BCVA in two out of three eyes between baseline and final visit. There were no signs of choroidal neovascularisation on OCT, FA, and/or ICGA.

The mean BCVA in Early Treatment of Diabetic Retinopathy Study letters was 71.2 ± 15.9 at last visit before PDT, which increased to 74.1 ± 14.1 at first visit after PDT (*p* = 0.093, compared with baseline) and 73.0 ± 19.1 at final visit (*p* = 0.392, compared with baseline). Multimodal imaging of a patient with a decrease in BCVA is depicted in Fig. 1. Mean CFT was 92.0 ± 26.0 μm at last visit before PDT, which increased to 98.1 ± 26.2 μm at first visit after PDT (*p* = 0.076), and changed to 97.0 ± 26.3 at final visit (*p* = 0.321). The mean SFCT in μm was 393.8 ± 112.2 at last visit before PDT (*n* = 23), which decreased to 345.0 ± 113.5 at first visit after PDT (*n* = 23; *p* = 0.032, compared with baseline). At final visit, the mean SFCT was 345.1 ± 113.4 μm (*n* = 25; *p* = 0.004, compared with baseline).

## Discussion

This study evaluated the outcome of half-dose PDT in cCSC eyes with pre-existing fovea-involving atrophy. A significantly higher percentage of eyes showed a complete resolution of SRF after half-dose PDT at final follow-up as compared with baseline. Moreover, after this treatment, the SFCT significantly decreased, and the EZ and ELM were significantly more often continuous at final visit, compared with baseline. However, no significant changes in BCVA and CFT were noted.

The relatively old age of the cCSC patients with fovea-involving atrophy and the relatively long disease duration and visual symptoms until PDT treatment in this study may imply a more prolonged presence of SRF in these cCSC eyes with fovea-involving atrophy. Long-standing disease has been previously associated with a worse BCVA outcome in cCSC [5, 14]. This presumed long-lasting detachment of the photoreceptor outer segments from the already damaged RPE can eventually cause irreversible damage to photoreceptor structure and function [17]. In an early stage of CSC, photoreceptor function and viability can be relatively maintained, potentially because of a partially preserved molecular exchange with the surrounding retinal structures, RPE and choroid, despite the presence of SRF between the photoreceptors and RPE. A reasonably adequate phototransduction and visual pigment regeneration may be possible through an alternative intraretinal visual cycle via Müller cells [18].

The percentage of cCSC eyes with fovea-involving atrophy with a complete resolution of SRF after half-dose PDT in this study (56% at final visit) was relatively low compared with previous studies on half-dose PDT in both general cCSC as well as a severe cCSC patient population (with described percentages of 67–100% complete resolution) [9, 11, 13, 19–21]. This indicates that half-dose PDT may be less effective in this specific cCSC subgroup with fovea-involving atrophy as compared with cCSC patients without these central atrophic changes. We found a significant decrease in the number of eyes with SRF after half-dose PDT, indicating that choroidal remodelling and—probably subsequent—SRF resolution induced by PDT is not necessarily hindered by the presence of foveal atrophy before treatment. However, the potential effects of PDT on the BCVA outcome may be limited especially in cCSC patients with fovea-involving atrophy, due to the presence of irreversible damage to the photoreceptors prior to treatment [5, 22]. This is also evident based on our observation that only 15% of patients had an intact foveal ELM before PDT, and only 9% had an intact EZ, which increased to 38% and 26%, respectively, after PDT. This structural improvement of the ELM and EZ after PDT may indicate that some degree of restoration of neuroretinal anatomy and function in this subgroup may occur.

This study is limited by its uncontrolled and retrospective nature, which has its implications in terms of availability of (long-term) outcome measurements. Because of the absence of a control group, our findings may not entirely be attributed to the treatment effects of PDT, but also to the natural disease course of cCSC with fovea-involving atrophy. Nevertheless, we were able to compare our findings with previous studies on PDT in cCSC. Retrospective studies tend to skew towards patients with worse outcomes because these patients are more likely to return, whereas patients who do well may be more often lost to follow-up. Although the available multimodal imaging and clinical characteristics were meticulously assessed, we cannot completely exclude the presence of a subtle flat sub-RPE (type 1) macular neovascularisation in a minority of the included eyes. In particular, in these cCSC patients with fovea-involving atrophy, OCT angiography would have been of added value in order to detect these neovascularisations. Moreover, no clear definition of fovea-involving atrophy in cCSC exists. We defined foveal atrophy as the presence of foveal hyperfluorescent abnormalities on FA due to an RPE window defect, which is usually accompanied by a decrease in CFT on OCT. The CFT reported in our study is comparable with the CFT previously reported in another study in cCSC patients with retinal atrophy, which has been found to be lower compared with cCSC patients in general [14]. However, clear cut-off points for the definition of foveal atrophy in cCSC would facilitate further research.

Half-dose (or half-fluence) is the preferred treatment in cCSC due to its superior efficacy in comparison with other treatment options, while it also has an excellent safety profile [11, 13]. In the current study, we show that half-dose PDT may also lead to a restoration of the EZ in cCSC with fovea-involving RPE atrophy. However, since extensive damage to the neuroretina and RPE is already present in cCSC with fovea-involving atrophy prior to PDT, the effect of half-dose PDT on clinical outcomes such as BCVA is limited. In cCSC patients with fovea-involving atrophy, half-dose PDT may be considered to restore neuroretinal anatomy and to potentially slow down disease progression, but patients should be adequately counselled on the more limited effects on BCVA. Future prospective studies with extensive phenotyping are warranted to shed a better light on the outcome and potential risks of PDT in this specific subgroup of cCSC with fovea-involving atrophy. At present, PDT appears to be the only effective and safe treatment for cCSC, at least for the general cCSC population, but its efficacy is more limited in advanced cCSC cases with foveal RPE atrophy and/or PCRD [10]. We therefore recommend that cCSC should ideally be treated with half-dose (or half-fluence) PDT before the development of PCRD or foveal atrophy and extensive RPE alterations [10, 11]. A quest for additional effective treatments, based on research on pathophysiology and potential new treatment targets and large prospective randomized controlled trials, is pivotal to broaden the therapeutic armamentarium for this mysterious disease.

## Data Availability

Data is available upon request.

## References

[1] Gass JD (1967). Pathogenesis of disciform detachment of the neuroepithelium: II. Idiopathic central serous choroidopathy. Am J Ophthalmol.

[2] Haimovici R, Koh S, Gagnon DR, Lehrfeld T, Wellik S (2004). Risk factors for central serous chorioretinopathy: a case-control study. Ophthalmology.

[3] Sakurada Y, Fragiotta S, Leong BCS, Parikh R, Hussnain SA, Freund KB (2019) Relationship between choroidal vascular hyperpermeability, choriocapillaris flow density, and choroidal thickness in eyes with pachychoroid pigment epitheliopathy. Retina (Philadelphia, Pa). doi:10.1097/IAE.0000000000002635 10.1097/IAE.0000000000002635 31415450

[4] Singh SR, Matet A, van Dijk EHC, Daruich A, Fauser S, Yzer S, Peiretti E, Sivaprasad S, Lotery AJ, Boon CJF, Behar-Cohen F, Freund KB, Chhablani J (2018). Discrepancy in current central serous chorioretinopathy classification. Br J Ophthalmol.

[5] Mrejen S, Balaratnasingam C, Kaden TR, Bottini A, Dansingani K, Bhavsar KV, Yannuzzi NA, Patel S, Chen KC, Yu S, Stoffels G, Spaide RF, Freund KB, Yannuzzi LA (2019). Long-term visual outcomes and causes of vision loss in chronic central serous chorioretinopathy. Ophthalmology.

[6] Breukink MB, Dingemans AJ, den Hollander AI, Keunen JE, MacLaren RE, Fauser S, Querques G, Hoyng CB, Downes SM, Boon CJ (2017) Chronic central serous chorioretinopathy: long-term follow-up and vision-related quality of life. Clinical ophthalmology (Auckland, NZ) 11:39-46. 10.2147/OPTH.S115685 10.2147/OPTH.S115685 PMC5189979 28053499

[7] van Rijssen TJ, van Dijk EHC, Scholz P, Breukink MB, Blanco-Garavito R, Souied EH, MacLaren RE, Querques G, Fauser S, Hoyng CB, Downes SM, Boon CJF (2019). Patient characteristics of untreated chronic central serous chorioretinopathy patients with focal versus diffuse leakage. Graefe's archive for clinical and experimental ophthalmology = Albrecht von Graefes Archiv fur klinische und experimentelle Ophthalmologie.

[8] van Rijssen TJ, van Dijk EHC, Scholz P, Breukink MB, Blanco-Garavito R, Souied EH, Keunen JEE, MacLaren RE, Querques G, Fauser S, Downes SM, Hoyng CB, Boon CJF (2019). Focal and diffuse chronic central serous chorioretinopathy treated with half-dose photodynamic therapy or subthreshold micropulse laser: PLACE trial report no. 3. Am J Ophthalmol.

[9] Mohabati D, van Dijk EH, van Rijssen TJ, de Jong EK, Breukink MB, Martinez-Ciriano JP, Dijkman G, Hoyng CB, Fauser S, Yzer S, Boon CJ (2018). Clinical spectrum of severe chronic central serous chorioretinopathy and outcome of photodynamic therapy. Clinical ophthalmology (Auckland, NZ).

[10] Mohabati D, Hoyng CB, Yzer S, Boon CJF (2019) Clinical characteristics and outcome of posterior cystoid macular degeneration in chronic central serous chorioretinopathy. Retina (Philadelphia, Pa). 10.1097/iae.0000000000002683 10.1097/IAE.0000000000002683 PMC7447130 31815880

[11] van Rijssen TJ, van Dijk EHC, Yzer S, Ohno-Matsui K, Keunen JEE, Schlingemann RO, Sivaprasad S, Querques G, Downes SM, Fauser S, Hoyng CB, Piccolino FC, Chhablani JK, Lai TYY, Lotery AJ, Larsen M, Holz FG, Freund KB, Yannuzzi LA, Boon CJF (2019). Central serous chorioretinopathy: towards an evidence-based treatment guideline. Prog Retin Eye Res.

[12] Daruich A, Matet A, Dirani A, Bousquet E, Zhao M, Farman N, Jaisser F, Behar-Cohen F (2015). Central serous chorioretinopathy: recent findings and new physiopathology hypothesis. Prog Retin Eye Res.

[13] van Dijk EHC, Fauser S, Breukink MB, Blanco-Garavito R, Groenewoud JMM, Keunen JEE, Peters PJH, Dijkman G, Souied EH, MacLaren RE, Querques G, Downes SM, Hoyng CB, Boon CJF (2018). Half-dose photodynamic therapy versus high-density subthreshold micropulse laser treatment in patients with chronic central serous chorioretinopathy: the PLACE trial. Ophthalmology.

[14] Wang MS, Sander B, Larsen M (2002). Retinal atrophy in idiopathic central serous chorioretinopathy. Am J Ophthalmol.

[15] Imamura Y, Fujiwara T, Spaide RF (2011). Fundus autofluorescence and visual acuity in central serous chorioretinopathy. Ophthalmology.

[16] van Rijssen TJ, Mohabati D, Dijkman G, Theelen T, de Jong EK, van Dijk EHC, Boon CJF (2018). Correlation between redefined optical coherence tomography parameters and best-corrected visual acuity in non-resolving central serous chorioretinopathy treated with half-dose photodynamic therapy. PLoS One.

[17] Cardillo Piccolino F, de la Longrais RR, Ravera G, Eandi CM, Ventre L, Abdollahi A, Manea M (2005). The foveal photoreceptor layer and visual acuity loss in central serous chorioretinopathy. Am J Ophthalmol.

[18] Wang JS, Kefalov VJ (2011). The cone-specific visual cycle. Prog Retin Eye Res.

[19] Fujita K, Imamura Y, Shinoda K, Matsumoto CS, Mizutani Y, Hashizume K, Mizota A, Yuzawa M (2015). One-year outcomes with half-dose verteporfin photodynamic therapy for chronic central serous chorioretinopathy. Ophthalmology.

[20] Lai FH, Ng DS, Bakthavatsalam M, Chan VC, Young AL, Luk FO, Tsang CW, Brelen ME (2016). A multicenter study on the long-term outcomes of half-dose photodynamic therapy in chronic central serous chorioretinopathy. Am J Ophthalmol.

[21] Lim JI, Glassman AR, Aiello LP, Chakravarthy U, Flaxel CJ, Spaide RF (2014). Collaborative retrospective macula society study of photodynamic therapy for chronic central serous chorioretinopathy. Ophthalmology.

[22] van Rijssen TJ, van Dijk EHC, Dijkman G, Boon CJF (2018). Clinical characteristics of chronic central serous chorioretinopathy patients with insufficient response to reduced-settings photodynamic therapy. Graefe's archive for clinical and experimental ophthalmology = Albrecht von Graefes Archiv fur klinische und experimentelle Ophthalmologie.

